# Selenium-dependent metabolic reprogramming during inflammation and resolution

**DOI:** 10.1016/j.jbc.2021.100410

**Published:** 2021-02-11

**Authors:** Arvind M. Korwar, Ayaan Hossain, Tai-Jung Lee, Ashley E. Shay, Venkatesha Basrur, Kevin Conlon, Philip B. Smith, Bradley A. Carlson, Howard M. Salis, Andrew D. Patterson, K. Sandeep Prabhu

**Affiliations:** 1 Department of Veterinary and Biomedical Sciences, The Pennsylvania State University, University Park, Pennsylvania, USA; 2 Bioinformatics and Genomics, The Pennsylvania State University, University Park, Pennsylvania, USA; 3 Departments of Chemical Engineering, Biological Engineering, and Biomedical Engineering, The Pennsylvania State University, University Park, Pennsylvania, USA; 4 Department of Pathology, Proteomics Resource Facility, University of Michigan Medical School, Ann Arbor, Michigan, USA; 5 The Huck Institutes of the Life Sciences, Metabolomics Facility, The Pennsylvania State University, University Park, Pennsylvania, USA; 6 Molecular Biology of Selenium Section, Mouse Cancer Genetics Program, NCI, National Institutes of Health, Bethesda, Maryland, USA

**Keywords:** macrophages, peritonitis, proteomics, redox, succinate dehydrogenase, BMDMs, bone marrow–derived macrophages, Carkl, carbohydrate-like kinase, DMM, dimethylmalonate, Hif-1α, hypoxia inducible factor-1α, Idh1, isocitrate dehydrogenase, IL-4, interleukin-4, LPS, lipopolysaccharide, OXPHOS, oxidative phosphorylation, Pkm2, pyruvate kinase 2, PPP, pentose phosphate pathway, Ri, resolution index, ROS, reactive oxygen species, Sdh, succinate dehydrogenase complex, Se, Selenium, Shpk, Sedoheptulokinase, TCA, tricarboxylic acid, TMT, tandem mass tag, Trsp, tRNA^[Ser]Sec^

## Abstract

Trace element selenium (Se) is incorporated as the 21st amino acid, selenocysteine, into selenoproteins through tRNA^[Ser]Sec^. Selenoproteins act as gatekeepers of redox homeostasis and modulate immune function to effect anti-inflammation and resolution. However, mechanistic underpinnings involving metabolic reprogramming during inflammation and resolution remain poorly understood. Bacterial endotoxin lipopolysaccharide (LPS) activation of murine bone marrow–derived macrophages cultured in the presence or absence of Se (as selenite) was used to examine temporal changes in the proteome and metabolome by multiplexed tandem mass tag–quantitative proteomics, metabolomics, and machine-learning approaches. Kinetic deltagram and clustering analysis indicated that addition of Se led to extensive reprogramming of cellular metabolism upon stimulation with LPS enhancing the pentose phosphate pathway, tricarboxylic acid cycle, and oxidative phosphorylation, to aid in the phenotypic transition toward alternatively activated macrophages, synonymous with resolution of inflammation. Remodeling of metabolic pathways and consequent metabolic adaptation toward proresolving phenotypes began with Se treatment at 0 h and became most prominent around 8 h after LPS stimulation that included succinate dehydrogenase complex, pyruvate kinase, and sedoheptulokinase. Se-dependent modulation of these pathways predisposed bone marrow–derived macrophages to preferentially increase oxidative phosphorylation to efficiently regulate inflammation and its timely resolution. The use of macrophages lacking selenoproteins indicated that all three metabolic nodes were sensitive to selenoproteome expression. Furthermore, inhibition of succinate dehydrogenase complex with dimethylmalonate affected the proresolving effects of Se by increasing the resolution interval in a murine peritonitis model. In summary, our studies provide novel insights into the role of cellular Se *via* metabolic reprograming to facilitate anti-inflammation and proresolution.

Trace element selenium (Se) is incorporated as the 21st amino acid, selenocysteine, *via* tRNA^[Ser]Sec^ (encoded by *Trsp*) dependent decoding of the UGA codon in 24 murine (25 in humans) selenoproteins (1, 2). Selenoproteins function as redox gatekeepers and catalyze reactions involving reduction of disulfides, methionine sulfoxide, and peroxides (1, 3). In addition, selenoproteins also modulate immune functions through oxidative homeostasis, prevention of iron-induced cellular ferroptosis, regeneration of reduced thioredoxin, regulation of actin repolymerization during innate immune response, and cellular calcium homeostasis (4). Although a highly debated topic, Se supplementation and its benefits in severe systemic inflammatory response syndrome, sepsis, and septic shock assumes a J-shaped curve relationship, suggesting supplementation therapies primarily benefit Se-deficient patients (5, 6). However, their mechanistic role in the modulation of pathways associated with metabolic reprograming during inflammation is poorly understood (7, 8, 9, 10, 11).

Phenotypic plasticity of macrophages as seen in the form of classically activated M1 proinflammatory phenotype, upon treatment with bacterial endotoxin lipopolysaccharide (LPS), or an alternatively activated (M2) anti-inflammatory phenotype, a trait synonymous with cellular mechanisms of resolution, upon treatment with interleukin (IL)-4 or IL-13, represents two ends of the polarization spectrum (12). The resolution program involves a highly coordinated and systemic response, involving transmigration, phagocytosis, antigen presentation, and expression of proresolving genes such as arginase-1 and Mrc-1 (CD206) that impinge on redox-dependent signaling and cellular metabolism (12, 13, 14, 15, 16). Monocyte/macrophage-specific deletion of selenoproteins has led us to recognize the importance of the selenoproteome in the transition of M1- to M2-like phenotype and resolution as seen in experimental models of gut inflammation and hematologic malignancies (17, 18, 19, 20, 21, 22, 23, 24, 25). Recent studies from our laboratory using tandem mass tag (TMT)-labeling method of nonselenocysteine peptides in murine bone marrow–derived macrophages (BMDMs) indicated a temporal regulation with a general increase in the selenoproteome upon LPS stimulation in a Se-dependent manner (26). Selenow, Gpx1, Msrb1, and Selenom were highly upregulated upon stimulation with LPS when compared with other selenoproteins. Together, it appears that selenoprotein-dependent pathways of anti-inflammation and proresolution likely impinge on metabolic reprogramming, which is not well understood.

It is evident that changes in intracellular metabolic pathways, such as glycolysis, pentose phosphate pathway (PPP), tricarboxylic acid (TCA) cycle, fatty acid oxidation, fatty acid synthesis, and amino acid metabolism, impact cellular functions in immune cells (27, 28). Macrophages stimulated with LPS predominantly display a glycolytic metabolic phenotype (14) and decreased oxidative phosphorylation (OXPHOS), like the “Warburg effect” in cancer cells (14, 17, 27, 29). LPS-activated macrophages inhibit expression of several enzymes of the TCA cycle, including succinate dehydrogenase (Sdh) complex (14) leading to accumulation of succinate and increased activation of hypoxia inducible factor-1α (Hif-1α) and IL-1β culminating in high glycolytic rates to favor M1 polarization (30). In fact, two distinct breaks have been identified in the TCA cycle in M1 macrophages that include the isocitrate dehydrogenase (Idh1) and Sdh complex (14, 31). Interruption of Idh1 and Sdh results in accumulation of citrate and itaconate (32, 33), and succinate, respectively. Conversely, M2 macrophages predominantly exhibit an OXPHOS metabolic phenotype (27, 28), relying on oxidation of glucose and fatty acids to sustain OXPHOS-mediated generation of ATP. Furthermore, LPS activation of macrophages downregulates pyruvate kinase 2 (Pkm2), whereas Pkm2 activation inhibits IL-1α production and Hif-1α–dependent genes, promoting an M2-like phenotype (34). In addition, the activity of sedoheptulokinase (Shpk) or carbohydrate-like kinase (Carkl), which catalyzes an orphan reaction in the PPP, involving the conversion of sedoheptulose to sedoheptulose 7-phosphate was shown to refocus cellular metabolism favoring an M2-like phenotype and a high-redox state (35).

Using TMT-labeled quantitative proteomics, targeted metabolomics, and unsupervised learning algorithms, we report here that Se in the form of selenoproteins modulates metabolic pathways involving the Pkm2, Shpk, and Sdh complex to predispose activated macrophages toward OXPHOS to efficiently regulate inflammation and timely resolution. Inhibition of the Sdh complex with dimethylmalonate (DMM) (36) affected the proresolving effects of Se in a zymosan model of peritonitis. Therefore, cellular Se, through its incorporation into selenoproteins, serves as a diet-derived regulator of metabolic reprograming to facilitate anti-inflammation and proresolution.

## Results

### Activation of macrophages and effect of Se supplementation on global proteome

**Figure 1:**
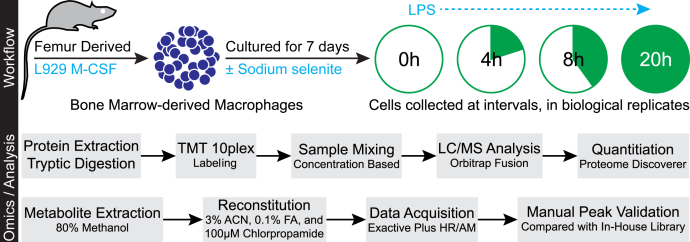
**Experimental design and data acquisition.** Bone marrow–derived macrophages (BMDMs) were harvested and cultured for 7 days either in the presence or absence of Se (as sodium selenite, Na_2_SeO_3_, 250 nM). BMDMs were stimulated with 100 ng of LPS for indicated time periods up to a maximum of 20 h. Cells were harvested after two washes with PBS and lysed to extract the proteome. Proteome was extracted and subjected to tryptic digestion followed by TMT labeling. Datasets were acquired on Lumos Fusion MS and analyzed by using Proteome Discoverer. Metabolites were extracted in methanol, dried, and reconstituted in the mobile phase with chlorpropamide as the internal standard. The dataset was acquired on Exactive Plus MS. Corresponding metabolite ion chromatograms were extracted using Xcalibur and inspected manually. LPS, lipopolysaccharide; TMT, tandem mass tag.

**Figure 2:**
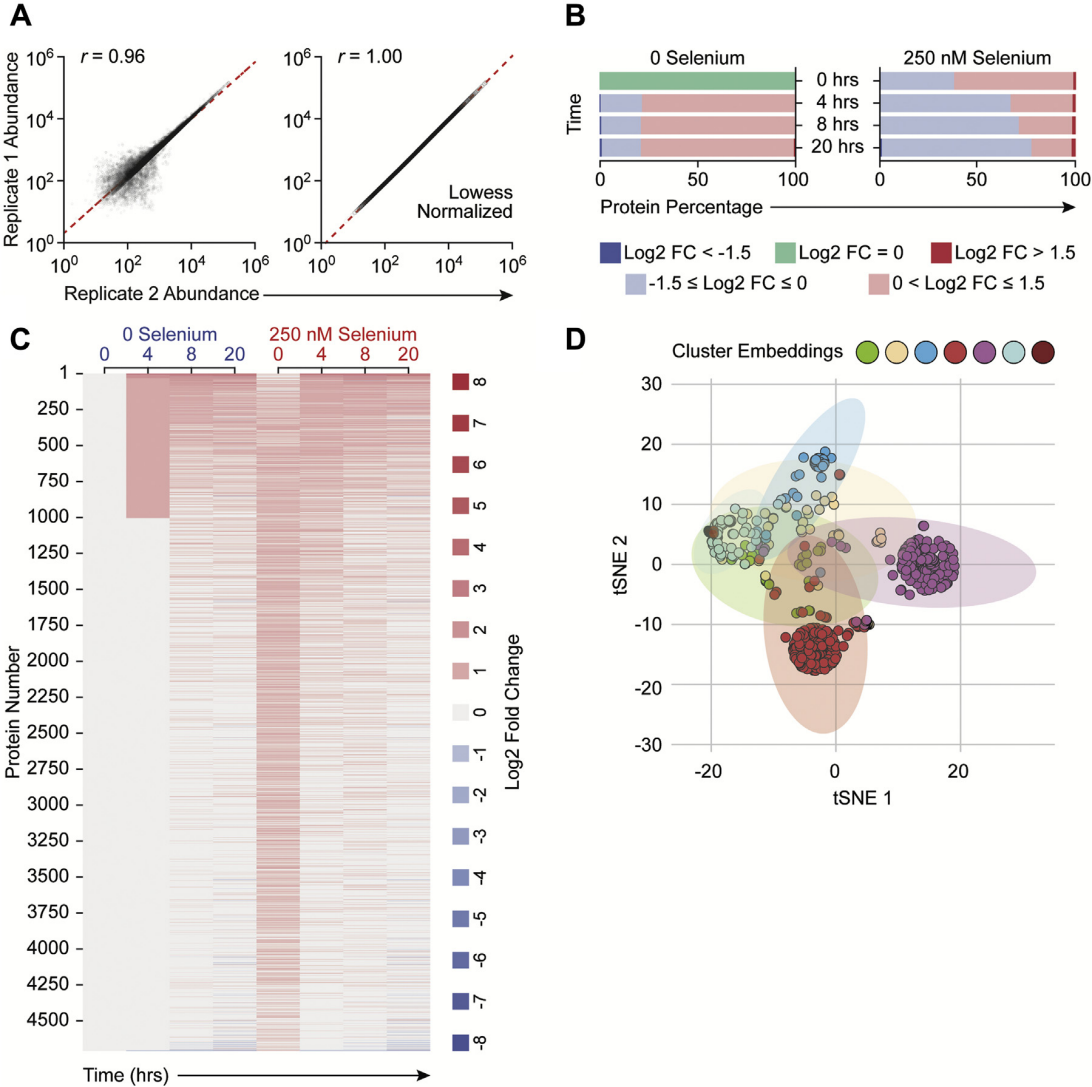
**Supplementation of macrophages with Se effects global proteomic changes upon treatment with LPS.** BMDMs were stimulated with LPS for indicated time periods up to a maximum of 20 h. Cells were harvested and lysed to extract the proteome. Proteomic analysis is described in Experimental procedures. BMDMs cultured in Se-deficient media and not subjected to any stimulation were used for comparison. Data were normalized to β-actin, and abundance values are expressed as relative to zero-hour zero-Se cells (naïve cells). *A*, scatterplots show biological replicates after LPS, which are well correlated (Pearson’s r of 0.96). *B*, the *bar chart* shows the overall proteome changes during after LPS stimulation. Values are represented as log2 fold changes. *C*, the heat map showing the modulation of global BMDM proteome after LPS stimulation. *D*, the t-SNE analysis and k-means clustering of proteomic changes after LPS show seven distinct clusters, respectively. BMDMs, bone marrow–derived macrophages; LPS, lipopolysaccharide; Se, selenium; t-SNE, t-distributed Stochastic neighbor embedding.

Based on the previously reported effects of Se and selenoproteins in favoring M2-like phenotype switching in macrophages (21), we comprehensively characterized the effects of Se on macrophage polarization after the temporal effects of LPS on differential proteome regulation in an effort to determine the underlying mechanisms by using a multiomics approach at various time points after LPS stimulation (Fig. 1). Murine BMDMs were polarized using LPS either in the absence or presence of Se as sodium selenite (250 nM). We first analyzed the proteomic dataset that indicated expression of prototypical LPS-responsive proteins (such as IL-1β, Nos2, Cd284, Stat1, and Icam-1), in agreement with previous literature (34) (Fig. S1). LPS-induced proteomic changes comprising 4710 proteins and their expression kinetics upon stimulation with LPS at 0, 4, 8, and 20 h were measured using tandem MS and showed very high correlation (Pearson’s *r* of 0.96 and 0.99) between biological replicates (Fig. 2*A*). Most importantly, LPS stimulation of Se-supplemented BMDMs led to temporal dynamic proteome regulation (Fig. 2*B*). Overall, ∼60% of the proteome was upregulated at 0 h, with log2 fold changes between 0 and 1.5, which eventually decreased to ∼32%, 28%, and 20% at 4, 8, and 20 h after LPS stimulation, respectively. In contrast, a larger percentage of proteins remained upregulated (>0 log2 fold change) in BMDMs treated without Se, throughout all time points. However, proteome upregulation (>1.5 log2 fold change) was observed only in cells treated with Se (Fig. 2*B*).

By applying clustering analysis of proteins with similar kinetics and magnitude of induction (Fig. 2*C*), we identified 121 proteins that were differentially regulated at 0 h in Se-treated cells compared with unstimulated Se-deficient BMDMs (“naïve” cells). Although majority of the selenoproteins were increased as reported earlier upon exogenous Se addition (26), nuclear factor-erythroid 2-related factor 2 target genes, such as thioredoxin reductase1 (Txnrd1) and Txnrd2, and hemeoxygenase 1 (Hmox1) were also increased by Se supplementation followed by LPS stimulation for 8 h. In contrast, 510 proteins were differentially regulated upon stimulation with LPS for 20 h (Fig. 2*C*). Interestingly, clustering analysis indicated heterogeneity within LPS-responsive proteins altered upon Se supplementation that separated into seven distinct clusters (Fig. 2*D*; Fig. S2). Specifically, clusters 1, 3, 4, 5, and 6 were enriched with 289, 631, 1292, 1496, and 536 proteins, respectively, upon LPS treatment (Fig. S2). Cluster 1 represented early induced proteins impacted between 4 and 8 h after LPS treatment. The magnitude of protein induction was higher in the presence of Se than 0 Se, which included proteins involved in the TCA cycle and electron-transport chain that were upregulated, which was further confirmed through pathway analysis. Significant enrichment of the TCA cycle and OXPHOS pathways in clusters 1, 5, and 6 showed induction of pathway-associated proteins from 4 h after LPS stimulation. Interestingly, we observed maximally expressed proteins in cluster 1, which corroborated the upregulation of proteins associated with the TCA cycle and OXPHOS (Fig. S2). Our analysis indicated that remodeling of metabolic pathways and consequent metabolic adaptation toward proresolving phenotypes started with Se treatment of Se-deficient BMDMs in the absence of any stimulation and became most prominent around 8 h after LPS stimulation.

### Se-dependent modulation of enzymes and metabolites in the TCA cycle, glycolysis, and PPP

Initiation and resolution of inflammation are energy-demanding processes that require timely adaptation of cellular metabolism for support. Because proteomic analysis suggested a plausible metabolic alteration in response to Se treatment, we examined the metabolic consequences of Se supplementation on macrophage activation.

**Figure 3:**
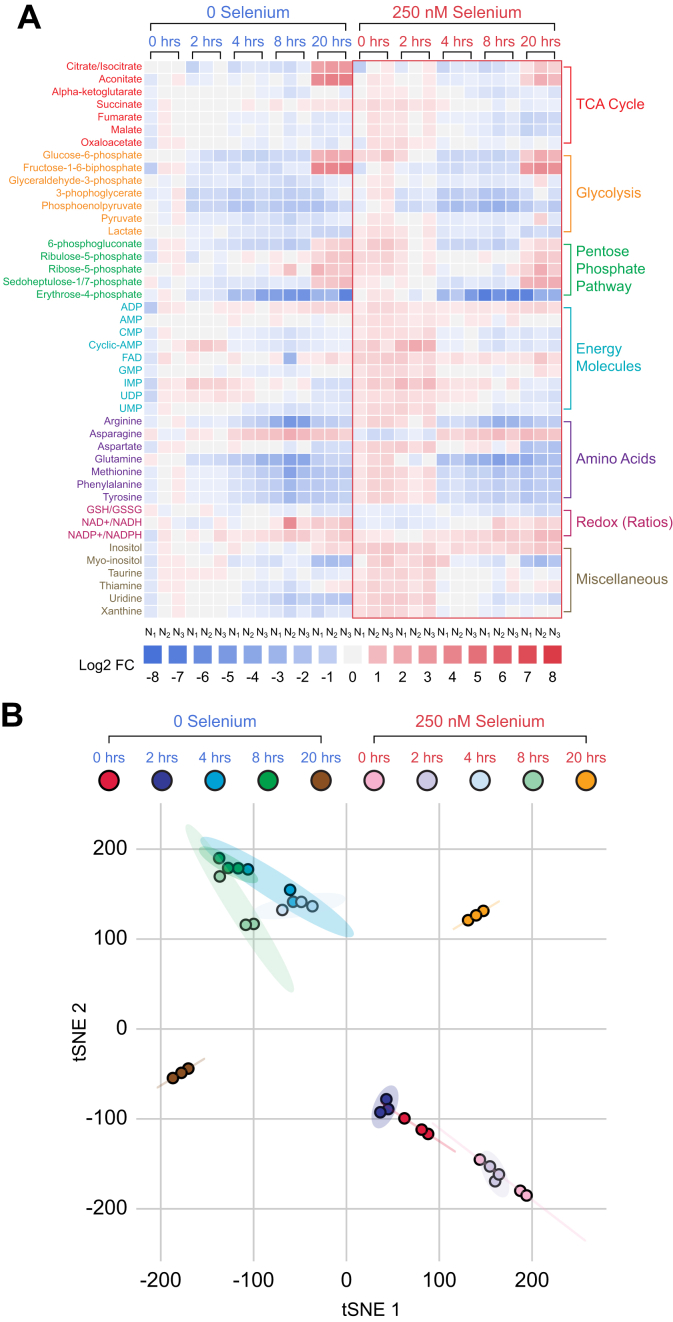
**Differential modulation of key metabolites *via* Se in LPS-stimulated BMDMs indicate a Se-dependent reduction in glycolytic pathway and increased the TCA cycle.** Metabolites were extracted in methanol, dried, and reconstituted in the mobile phase aqueous methanol containing 100-μM chlorpropamide as an internal standard. All samples were acquired in biological triplicate and randomized before 10 μl was acquired in LC-MS using reverse-phase UHPLC coupled to an Exactive Plus orbitrap MS. A total of 47 metabolites were profiled in biological triplicates. Individual metabolite extracted-ion chromatograms were used, with the peak area determined. All peak areas were normalized to internal standard chlorpropamide, and abundance was calculated relative to that of zero-hour zero-selenium cells (naïve cells). *A*, the heat map profiling of inflamed BMDMs show log2 fold change values of all 47 metabolites. All displayed log2 fold changes are represented in biological triplicates. *B*, the t-SNE method applied to all samples show distinct clusters separating the samples by time after LPS stimulation. BMDMs, bone marrow–derived macrophages; LPS, lipopolysaccharide; Se, selenium; TCA, tricarboxylic acid; t-SNE, t-distributed Stochastic neighbor embedding.

Intracellular metabolites were examined in BMDMs cultured either in the presence or absence of Se followed by stimulation with LPS for 2, 4, 8, and 20 h. LPS treatment profoundly affected a wide variety of intracellular metabolites as depicted in Figure 3*A*. t-distributed Stochastic neighbor embedding analysis indicated that LPS treatment groups were notably separated from each other (Fig. 3*B*). An interesting trend was observed for all time points after LPS treatment in cells cultured in the absence and presence of Se, where 0- to 8-h intervals were closest to each other. However, they were also most separated with respect to the presence or absence of Se. This difference was more profound at 20 h after LPS treatment between the two groups, further emphasizing the contribution of Se in reprograming of metabolites involved in the TCA cycle, glycolysis, PPP, energy molecules, redox molecules, and amino acids implicated in resolution of inflammation (Fig. 3*A*). Based on these studies, a targeted metabolomics approach was adopted to further investigate and compare the association between the TCA cycle, glycolysis, and PPP, upon stimulation with LPS.

**Figure 4:**
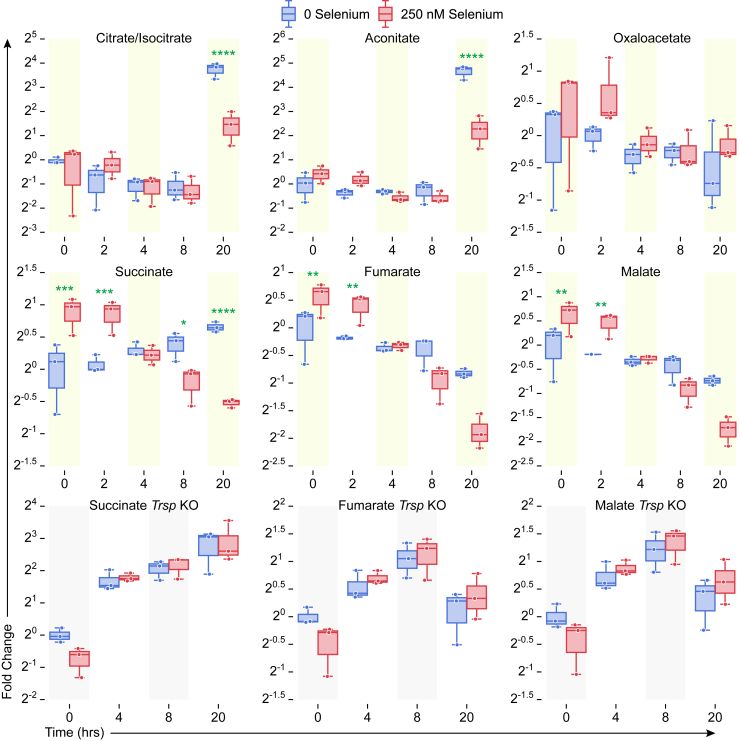
**Selenoprotein-dependent changes in the TCA cycle metabolites in WT and *Trsp***^***fl/f***^***LysM***^**Cre**^**BMDMs after LPS stimulation.** Metabolites were extracted from BMDM cells isolated from WT and *Trsp*^fl/f*l*^*LysM*^Cre^ mice as described earlier and analyzed by LC-MS. BMDMs from n = 3 mice per genotype were cultured as described earlier and stimulated for various time points 0 to 20 h with LPS. Metabolite peak areas were normalized to internal standard chlorpropamide, and abundance was calculated relative to that of zero-hour zero-selenium cells (naïve cells). Se supplementation of WT BMDMs with 250-nM Se (as selenite) showed dramatic regulation of succinate levels after LPS stimulation when compared with the BMDMs isolated from *Trsp*^*fl/fl*^*LysM*^*Cre*^ mice. BMDMs, bone marrow–derived macrophages; LPS, lipopolysaccharide; Se, selenium; TCA, tricarboxylic acid. ∗*p* < 0.05; ∗∗*p* < 0.005; ∗∗∗*p* < 0.0005; ∗∗∗∗*p* < 0.00005.

We first examined citrate/isocitrate levels in BMDMs cultured with or without Se, before and after LPS treatment. Untreated cells failed to show any temporal differences as in cells stimulated with LPS for the first 8 h. However, prolonged treatment for 20 h with LPS significantly increased citrate/isocitrate levels in Se-deficient cells when compared with macrophages treated with Se (Figs. 3*A* and 4). In addition, aconitate modulation followed a similar trend, with an increase at 20 h after LPS in Se-deficient cells (Figs. 3*A* and 4), suggesting a constant flux of citrate through the TCA cycle. Temporal modulation of succinate in response to exogenous Se treatment before and after LPS stimulation was interesting. Succinate levels were high in unstimulated cells in the presence of Se (compared with those without Se) that showed a gradual decline upon LPS stimulation starting at 4 h that persisted even at 20 h (Figs. 3*A* and 4). On the other hand, succinate levels increased in Se-deficient cells gradually over the 20 h after LPS treatment. Furthermore, fumarate, a product of succinate oxidation, and its hydrated product, malate, followed an identical pattern to that of succinate (Fig. 4). Interestingly, LPS-dependent temporal decrease in succinate, fumarate, and malate was not observed in BMDMs derived from *Trsp*^*fl/fl*^*LysM*^*Cre*^ *mice*, despite the presence of 250-nM selenite (Fig. 4) clearly implicating a unique role for Se/selenoproteins in ensuring an uninterrupted TCA cycle.

**Figure 5:**
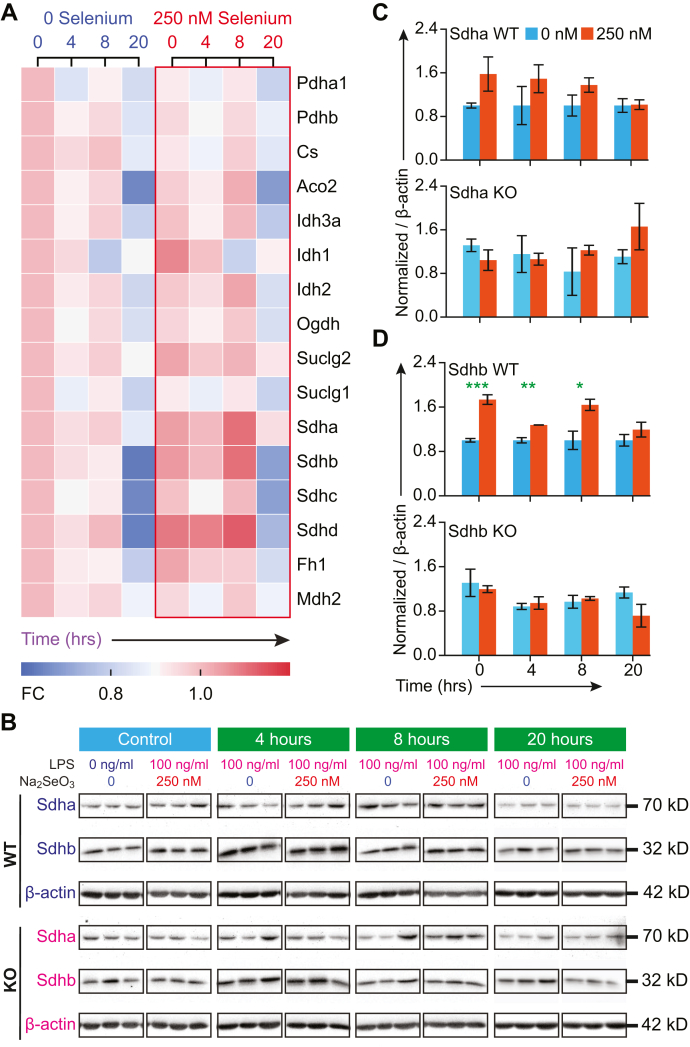
**Selenoprotein-dependent changes in the TCA cycle proteins.***A*, the heat map showing temporal regulation of the TCA cycle proteins as determined by proteomic studies. *B*, the Western blot of Sdha and Sdhb. BMDMs were isolated from WT and *Trsp*^*fl/fl*^*LysM*^*Cre*^ mice, respectively, and incubated with L929 containing DMEM without Na_2_SeO_3_ (250-nM Se) for 8 days. Cells were stimulated with 100-ng LPS on day eight for 1 h, and cells were harvested at 4, 8, and 20 h, followed by protein extraction and Western blot analysis of BMDMs in biological triplicate per genotype. Densitometric analysis of Sdha and Sdhb expression was performed using ImageJ and normalized to that of β-actin. *C* and *D*, densitometric evaluation of Western immunoblots for the expression of Sdha and Sdhb in BMDM incubated with Se compared with those cultured without Se before and after LPS stimulation. Representative data shown are the mean ± SEM of n =3 per genotype at each time point after LPS treatment. ∗*p* < 0.05; ∗∗*p* < 0.005; ∗∗∗*p* < 0.0005. BMDMs, bone marrow–derived macrophages; DMEM, Dulbecco’s Modified Eagle Medium; LPS, lipopolysaccharide; Se, selenium.

Given the Se-dependent decrease in succinate, we further examined the modulation of proteins associated with the TCA cycle. Notably, protein levels of Idh1 and individual subunits of the Sdh complex were higher in Se-supplemented BMDMs (Fig. 5). The mitochondrial proteome was classified into six clusters based on the kinetic regulation of protein expression upon LPS stimulation (Fig. S3). Western blot analysis of Sdha (flavoprotein subunit of complex II) corroborated proteomic dataset showing higher levels of expression at 0, 4, and 8 h after LPS stimulation (Fig. 5, *A*–*C*). However, expression of Sdha in *Trsp* KO BMDMs was decreased at time points 0, 4, and 8 h after LPS but increased at 20 h in the presence of 250-nM selenite treatment, indicating an opposite effect that corroborated the modulation of cellular succinate levels (Fig. 5, *B* and *C*). Western blot analysis of the expression of Sdhb (iron–sulfur subunit of complex) was higher in the WT BMDMs than BMDMs from *Trsp* KOs cultured with selenite (Fig. 5, *B* and *D*). In addition, fumarate hydratase (fumarase; Fh1) also increased in LPS-stimulated BMDMs cultured with Se, while malate dehydrogenase 2 displayed an increase at 4 h after LPS stimulation in Se-supplemented BMDMs compared with the Se-deficient counterpart (Fig. 5*A*). Interestingly, flow cytometry–based reactive oxygen species (ROS) measurements indicated cytosolic ROS levels were higher in Se-deficient cells that decreased after a transient increase with LPS stimulation followed by an increase at 20 h compared to Se-supplemented cells where the levels were held steady (Fig. S4*A*). However, Se-supplemented cells displayed higher levels of mitochondrial ROS (predominantly superoxide) (Fig. S4*B*), suggestive of an active TCA cycle and OXPHOS, which corroborates our proteomic and metabolomic studies.

**Figure 6:**
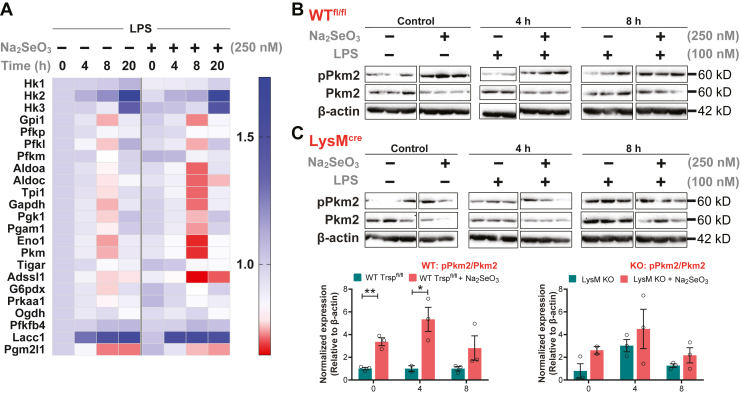
**Selenoprotein-dependent changes in glycolytic cycle proteins.***A*, the heat map showing temporal changes in glycolytic pathway proteins as determined by proteomic studies. *B* and *C*, the Western blot of pPkm2 and Pkm2 (total). BMDMs were isolated from WT and *Trsp*^fl/fl^*LysM*^Cre^ mice. Cells were stimulated with 100-ng LPS on day eight for 1 h and harvested at 4, 8, and 20 h, followed by protein extraction and Western blot analysis. Densitometric analysis of Western immunoblots using ImageJ program provided pPkm2:Pkm2 ratio that was normalized to β-actin and compared with cells cultured without Se. Representative data shown are the mean ± SEM of n =3 per genotype at each time point after LPS stimulation. ∗*p* < 0.05; ∗∗*p* < 0.005. BMDMs, bone marrow–derived macrophages; LPS, lipopolysaccharide; Se, selenium.

To further examine if Se supplementation of BMDMs impacted key glycolytic pathway metabolites before or after LPS stimulation, we examined the temporal modulation of the following metabolites: glucose 6-phosphate, fructose 1,6-bisphosphate, glyceraldehyde 3-phosphate, 3-phosphoglycerate, phosphoenolpyruvate, pyruvate, and lactate. These glycolytic metabolites showed varying levels of modulation in Se-deficient and Se-supplemented cells before and after LPS stimulation. Levels of glucose 6-phosphate, glyceraldehyde-3-phosphate, 3-phosphoglycerate, pyruvate, and lactate were higher in Se-supplemented macrophages than their Se-deficient counterparts before stimulation. Upon stimulation with LPS, a time-dependent decrease in these metabolites was observed in Se-supplemented cells, especially at 8 h after LPS (Fig. 3*A*, Fig. S5). In contrast to WT BMDMs, unstimulated BMDMs isolated from *Trsp* KO mice showed low levels of phosphoenolpyruvate and pyruvate that remained unchanged upon addition of sodium selenite (250 nM). LPS stimulation of BMDMs lacking selenoproteins showed a markedly different temporal regulation of these metabolites, in contrast to WT BMDMs, highlighting an important role for selenoproteins in cellular metabolism during inflammation (Fig. S5). Furthermore, proteomic expression profile of glycolytic enzymes showed generally reduced levels in Se-supplemented BMDMs compared with the Se-deficient BMDMs before and/or after stimulation with LPS (Fig. 6*A*). Gpi, Pfk-l, Aldoa, Aldoc, Tim, Gapdh, Pgk1, Pgm2, Eno1, and Pkm were all decreased at 8 h after LPS stimulation in Se-supplemented BMDMs compared with to Se-deficient counterparts (Fig. 6*A*). Particularly, Eno1 and Pkm (Pkm2) were drastically decreased in Se-supplemented BMDMs after 8-h LPS stimulation. The phosphorylated (and dimeric) form of Pkm exhibited an increase at 0, 4, and 8 h after LPS treatment in the presence of Se, whereas an opposite effect was seen in the absence of Se. Interestingly, *Trsp* KO BMDMs cultured in the presence of Se (250 nM) showed similar trends to the Se-deficient WT BMDMs with regard to phosphorylated Pkm2 (Fig. 6, *B* and *C*).

**Figure 7:**
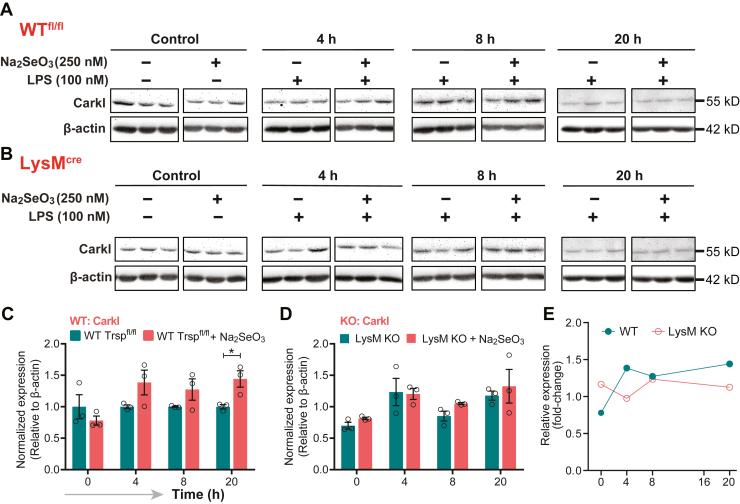
**Selenoprotein-dependent changes in Shpk (Carkl).***A* and *B*, western immunoblot of Carkl expression upon stimulation of BMDMs with LPS in the presence or absence of Se. BMDMs were isolated from WT and *Trsp*^fl/fl^*LysM*^Cre^ mice, respectively, and cells were stimulated with 100-ng LPS on day eight for 1 h and harvested at 4, 8, and 20 h, followed by protein extraction and Western blot analysis. *C* and *D*, Densitometric analysis using ImageJ of Shpk (Carkl) expression that was normalized to the expression of β-actin. Shpk fold change in BMDMs incubated with Se were compared with those without Se. *E*, fold change in relative expression of Shpk in WT and *LysM* KO over time post LPS stimulation. Representative data shown are the mean ± SEM of n =3 per genotype at each time point after LPS stimulation. ∗*p* < 0.05. BMDMs, bone marrow–derived macrophages; LPS, lipopolysaccharide; Se, selenium.

Key PPP metabolites were analyzed to examine the impact of changes in cellular Se status before or after LPS stimulation. 6-Phosphogluconate, ribulose 5-phosphate, ribose 5-phosphate, and erythrose 4-phosphate levels decreased upon Se supplementation, especially at 8 h after LPS treatment (Fig. 3*A*, Fig. S6). Interestingly, Shpk (Carkl), a key enzyme that catalyzes the formation of sedaheptulose-7-phosphate, was downregulated in Se-deficient BMDMs when compared with those cultured in the presence of Se. Accordingly, sedoheptulose 7-phosphate was higher in Se-supplemented BMDMs, particularly at 0 to 2 h, and later at 20 h after LPS treatment, suggesting that Se affects expression regulation of Shpk (Carkl) (Fig. 3*A*, Fig. S6). Western blot analysis of Shpk expression indicated modulation in WT BMDMs treated with Se starting at 4 h with differences persistent up to 20 h following LPS treatment. Such modulations were absent in *Trsp* KO BMDMs, suggesting that the effect of Se is mediated through selenoproteins (Fig. 7, *A* and *B*). Collectively, our results lend credence to the hypothesis that Se supplementation of BMDMs increases mobilization of metabolites *via* the PPP to potentially assist in various cellular functions to effect resolution, a thermodynamically active process.

### DMM treatment negatively impacts Se-dependent resolution of inflammation

**Figure 8:**
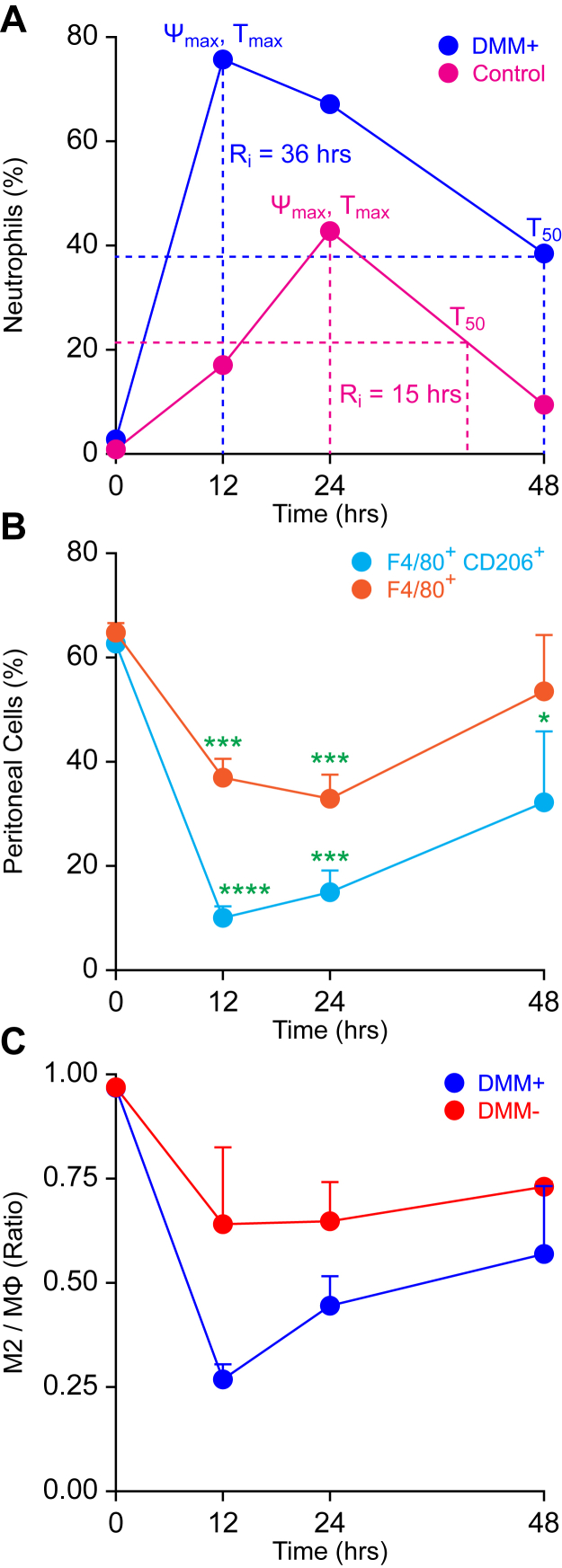
**DMM treatment of Se-Supplemented mice decreases the resolution index in a Zymosan-induced peritonitis model.** Se-supplemented mice were treated intraperitoneally with or without DMM in PBS (160 mg/kg of body weight) or PBS 3 h before administration of Zymosan A (10 mg/kg body weight) intraperitoneally. Mice were euthanized at 0, 12, 24, and 48 h after Zymosan A injection, and peritoneal exudates were collected, centrifuged at 400*g* for 5 min at 4 °C, washed with the flow buffer (PBS containing 2% FBS and 100 I.U. penicillin and 100 μg/ml streptomycin), resuspended in 1-ml flow buffer, and counted by trypan blue, and 100,000 viable cells were analyzed by flow cytometry. *A*, the resolution index (Ri) calculation based on the percentage of neutrophils (Ly6-G+ cells) in the peritoneal lavage fluid at each time point. Ψmax and Tmax represent the highest percentage of cells after Zymosan treatment and the time point at which it occurs, respectively. Data shown are representative of biological triplicates. *B*, the percentage of total macrophages (F4/80^+^) and M2-like macrophages (F4/80^+^ CD206^+^) in peritoneal lavage fluid as a function of time after Zymosan A treatment. *C*, the ratio of the total macrophages to M2-like macrophages in the peritoneal lavage fluid as a function of time after Zymosan A treatment. Data shown are the mean ± SEM of n = 3 to 4 per treatment group; ∗∗∗*p* < 0.0005. ∗∗∗∗*p* < 0.0001. DMM, dimethylmalonate; FBS, fetal bovine serum; Se, selenium.

Given that our data indicated Se supplementation led to decreased succinate levels in addition to the modulation of Sdh expression, we examined if inhibition of succinate oxidation in mice negatively impacted the effect of Se in the resolution of zymosan-induced peritonitis. Mice on a Se-supplemented diet (0.4 ppm) were treated intraperitoneally with either vehicle control (PBS) or DMM (160 mg/kg) in PBS, a competitive inhibitor of Sdh, 3 h before Zymosan A injection in a resolution index (Ri) experiment (Experimental procedures). DMM treatment followed by Zymosan A activation led to a higher percentage of neutrophils (∼75%) with an Ri = ∼36 h, whereas PBS-treated mice had ∼40% neutrophils and an Ri = ∼15 h (Fig. 8*A*). DMM treatment also led to a slight increase in total F4/80^+^ macrophages in response to Zymosan A stimulation (Fig. 8*B*), while decreasing the M2-like macrophages (F4/80^+^ CD206^+^) in the peritoneal lavage fluid (Fig. 8*C*) compared to the PBS control. These studies further corroborate *in vitro* observations and suggest that a Se-dependent increase in succinate oxidation is key to timely resolution of inflammation.

## Discussion

Resolution of inflammation is an active and highly orchestrated process that restores normal functional tissue homeostasis (37), where macrophages play an integral role (11, 12). Plasticity of these cells arises from their ability to skew metabolism from glycolysis and fatty acid synthesis in M1 macrophages to OXPHOS and fatty acid β-oxidation, in M2 macrophages, to meet their energy demands for survival and functionality (27). Here, using multiomics platforms, we examined the effect of micronutrient Se and selenoproteins on the reprogramming of metabolic pathways involving the Pkm, Shpk (Carkl), and Sdh complex to predispose macrophages toward OXPHOS and eventually facilitate resolution of LPS-induced inflammation.

In addition to increased mitochondrial ROS levels, significant reduction in both citrate and aconitate, along with increased Idh expression, decreased α-ketoglutarate, succinate, fumarate, malate, and oxaloacetate by exogenous Se suggested increased mobilization of the TCA cycle, when compared to Se-deficient cells. Temporal increase in succinate after LPS, which moonlights as a key mediator of inflammation (30), was reversed by Se with an increase in fumarate and malate. Inhibition of the Sdh complex with DMM (36) affected the proresolving effects of Se in Zymosan-induced peritonitis in mice. Although the exact mechanisms of regulation of the Sdh complex by selenoproteins are unclear, inhibition of succination of reactive cysteine in fumarate hydratase (38) by Se could serve as a potential mechanism to help restore the broken TCA cycle.

Surprisingly, despite the increase in succinate that is known to increase Hif-1α, the expression of Hif-1α or its characteristic downstream genes, including IL-1β, glycolytic enzymes, and glucose transporters (30), was not observed in Se-deficient cells. However, we observed a decrease in glycolytic enzymes in macrophages supplemented with Se, suggesting considerable reduction in glycolytic rates. Pkm, a key regulatory node, was found to be significantly downregulated in macrophages supplemented with Se. Pkm is also regulated by phosphorylation status by upstream kinases that is indicative of dimer/tetramer formation (39). The dimeric and phosphorylated forms of Pkm2, which are enzymatically inactive, also positively regulate their expression and that of Hif-1α–dependent genes (40). The effects we observed were not similar to that reported with DASA-58, a selective activator of Pkm2, which inhibited the Hif-1α–dependent induction of glycolytic proteins by LPS (34), suggesting Hif-1α–independent control of metabolic effects in the presence of Se. Along with PKM, there was a decrease in the expression of Pfkm and Pfkl, but an increased AMP:ADP ratio in BMDMs cultured with Se, along with other nucleotides that persisted even after LPS stimulation (Fig. 3*A*). Together, such changes indicated functional glycolysis, albeit at low levels, necessitating auxiliary pathways such as the PPP that utilize glycolytic intermediates to produce precursors of NADPH, along with increased selenoprotein expression, to maintain redox homeostasis and help buffer ROS to ultimately influence macrophage polarization (28).

Previous studies suggested that loss of Shpk (Carkl) led to a significant drop of NADH levels resulting in a redox shift (35). Compared to the Se-deficient cells, Se supplementation of BMDMs held the NAD^+^:NADH ratio up to 4 h after LPS treatment, whereas NADP^+^:NADPH ratios were unchanged. The GSH:GSSG ratio decreased temporally in Se-supplemented macrophages upon LPS stimulation but tightly maintained in Se-supplemented cells, suggesting restoration of the redox status in favor of resolution. Increased levels α-ketoglutarate, aspartate, and glutamine in Se-supplemented cells corroborated with restoration of GSH/GSSG balance and the TCA cycle through increased α-ketoglutarate. Surprisingly, Txrnd1 and Hmox1 were mostly seen to increase transiently with Se, suggesting a fine control by nuclear factor erythroid 2–related factor 2–dependent mechanisms.

Se treatment of BMDMs increased cellular levels of amino acids before and after LPS (up to 2 h), perhaps to support many functions of macrophages in addition to metabolic reprogramming. Although not exactly identical, a similar trend was reported earlier in the whole liver (41). In addition, Se also increased monocarboxylate transporters, Slc16a1 and Slc16a3, and a high-affinity copper transporter Slc31a1, to perhaps support increased pyruvate metabolism, TCA cycle, and OXPHOS (data not shown). Macronutrients such as *myo*-inositol, a precursor for synthesis of phosphoinositides critical for signal transduction *via* second messengers IP3 and Ca^2+^, were consistently higher in the presence of Se and both in the absence or presence of LPS. Thus, a broader understanding of Se on *myo*-inositol metabolism in regulating macrophage functions may benefit from further studies.

Given the importance of fatty acid oxidation and OXPHOS, which drives the (M2) macrophage-mediated resolution and tissue repair (42), higher expression of Acsl3 and Acsl4, which are involved in fatty acid metabolism, along with Acadl, Acsl5, Adam17, Cyb5r3, Gcdh, Sgpp1, Sptlc1, Sptlc2, and the acetylCoA transporter slc33a1 (proteins represented by gene names), was temporally upregulated by Se (data not shown). In agreement with Se-dependent endogenous activation of PPARγ as reported previously (19), which is implicated in regulation of M2 genes, including Arg-1 and Mrc-1, fatty acid oxidation, adipocyte differentiation, and glucose homeostasis were upregulated upon LPS treatment (19, 43, 44, 45). Pathway network analysis suggested Ppar signaling–associated proteins such as Csf2rb, Cbl, Akt3, Pik3r5, Stat3, and Irf9 were upregulated by Se supplementation. Stat3 mediates the anti-inflammatory effects of IL-10 (46), further reinforcing the anti-inflammatory role of Se *via* multiple mechanisms that promote the proresolution phenotype of BMDMs.

**Figure 9:**
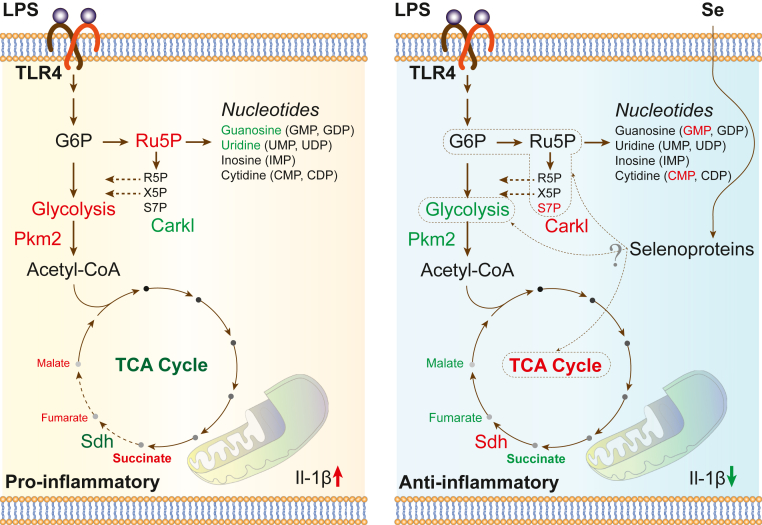
**Schematic representation illustrating the effect of Se on BMDMs before and after LPS stimulation.** Key metabolites and pathways that were significantly upregulated (*red*) or downregulated (*green*) in LPS-treated (100 ng/ml) BMDMs ± Se (250 nM) are shown. BMDMs, bone marrow–derived macrophages; LPS, lipopolysaccharide; Se, selenium.

In conclusion, our studies demonstrate a key role for micronutrient Se in the form of selenoproteins in resolution, as seen in *in vitro* and *in vivo* models of inflammation. Although the exact mechanistic underpinnings are still not clear, it is evident that exogenous addition of Se to Se-deficient BMDMs regulates key metabolic nodes, Sdh complex, Pkm2, and Shpk (Carkl), in inflamed macrophages to promote anti-inflammation and resolution of inflammation (Fig. 9). Although these studies provide some explanation as to why Se-deficient patients with sepsis respond better to Se therapy, our results further reiterate the importance of maintaining cellular Se, which tends to decrease in the elderly or those with chronic inflammatory disorders, to be important for effective resolution of inflammation.

## Experimental procedures

### Materials

The TMT-labeling reagent kit and Dulbecco’s Modified Eagle Medium were procured from Thermo Fisher Scientific. Sodium selenite and LPS from *Escherichia coli* (Serotype 0111:B4) were procured from Sigma Aldrich. Fetal bovine serum and L929 fibroblasts were purchased from Atlanta Biologicals and the American Type Culture Collection, respectively. The basal level of Se in the culture media was 7 nM as determined by inductively coupled plasma mass spectrometry–based methods. All other chemicals and reagents were of MS grade.

### Mice and genotyping

Three-week-old C57Bl/6 male mice were purchased from Taconic Biosciences, Inc and maintained on either an AIN-76–based semipurified Se-deficient diet (<0.01 ppm) or Se-supplemented diet (0.4 ppm) from Harlan-Teklad for at least 4 weeks before being used in the experiments as described previously (21). A transgenic C57Bl/6 line carrying a lysozyme M Cre (*LysM*^Cre^) transgene was crossed to floxed *Trsp* (*Trsp*^*fl/fl*^) allele, both generously provided by Dr Dolph Hatfield (the NIH, Bethesda, MD), as described previously (47). Genotyping was performed as described previously (21). All studies were preapproved by the Institutional Animal Care and Use Committee and the Institutional Biosafety Committee at the Pennsylvania State University (University Park, PA).

### BMDM culture: LPS stimulation

Three-week-old male C57Bl/6 mice were purchased from Taconic Biosciences, Inc and maintained on either an AIN-76–based semipurified Se-deficient diet (<0.01 ppm; Se-D) or Se-supplemented diet (0.4 ppm as selenite; Se-S) purchased from Harlan Teklad, for 7 weeks before being used in the experiments. Femoral bone marrow from Se-D and Se-S mice were used as a source of BMDMs upon culture with no exogenous or 250-nM Se (as sodium selenite) addition, respectively, as described (26). Cells were cultured in biological triplicates for 7 days with or without Se (as sodium selenite; 250 nM) as described previously (19, 26). On the eighth day, cells were stimulated with LPS (100 ng/ml; Sigma), where cells were treated with LPS for an hour followed by replacement with fresh culture media until harvest at 0, 4, 8, and 20 h after stimulation. Cells were washed with ice-cold sterile PBS and scraped, and cell pellets were stored at −80 °C until further processing.

### Murine peritonitis and flow cytometry

Peritoneal inflammation was induced as described previously (48, 49). Se-S mice were treated intraperitoneally with DMM in PBS (160 mg/kg of body weight) or PBS alone 3 h before administration of Zymosan A (10 mg/kg body weight) intraperitoneally. Mice were euthanized at 0, 12, 24, and 48 h after Zymosan A injection, and peritoneal exudates were collected, centrifuged at 400*g* for 5 min at 4 °C, washed, and resuspended in the flow buffer (PBS containing 2% fetal bovine serum; 1 ml), and 100,000 viable cells were analyzed by flow cytometry. Pellets were suspended in 100 μl of the flow buffer and blocked with 0.25-μg Fc block (BD Pharmingen) for 10 min followed by staining with antibodies: 1 μl of phycoerythrin-conjugated anti-mouse Ly-6G (BD Pharmingen), 1 μl of FITC-conjugated anti-mouse CD206 (BioLegend), and 10 μl of APC-conjugated anti-mouse F4/80 (Miltenyi Biotec) for 30 min at 4 °C in the dark. Cells were washed twice with the flow buffer and centrifuged at 400*g* for 5 min at 4 °C and analyzed on a BD Accuri C6 Flow Cytometer and analyzed with FlowJo, version 10, software (FlowJo, LLC). The resolution of inflammation defined as the Ri is the time taken to reduce the number of neutrophils at the site of inflammation by 50% (50). All animal protocols were preapproved by the Institutional Animal Care and Use Committee at the Penn State University.

### Sample preparation for proteomics, data acquisition, and analysis

The proteome was extracted from frozen cell pellets in RIPA buffer (Thermo Fisher Scientific), and the protein concentration was determined by the bicinchoninic acid assay (Thermo Fisher Scientific). Equal amounts of protein (100 μg) were diluted with 100-mM triethyl ammonium bicarbonate buffer and reduced with dithiothreitol (10 mM) at 60 °C for 30 min, followed by alkylation with 2-chloroacetamide (65 mM) at room temperature in the dark for 30 min. The proteome was digested with proteomic grade tosyl phenylalanyl chloromethyl ketone–trypsin at 1:50 (enzyme-to-substrate) ratio overnight at 37 °C. The peptide digest was labeled with TMT and fractionated (lot number SH253273, and sample to TMT channel information is provided in Table S1). The proteomic dataset was acquired on samples in biological triplicate using an UltiMate 3000 RSLCnano system coupled online to an Orbitrap Fusion MS (Thermo Fisher Scientific) as described previously (26). Briefly, peptides were resolved on an Acclaim PepMap C18 column (2 microns, 75 μm i.d. × 50 cm) with a flow rate of 300 nl/min using a 0.1% formic acid/acetonitrile gradient. For MultiNotch MS3, the top ten precursors from each MS2 scan were fragmented by higher-energy collisional dissociation followed by Orbitrap analysis (NCE 55; 60,000 resolution; AGC 5 × 104; max IT 120 ms, 100–500 m/z scan range). Mass spectral datasets were analyzed with Proteome Discoverer (v2.2, Thermo Fisher Scientific) using SEQUEST-HT algorithm for database search for peptide identification and queried against the UniProt *Mus Musculus* database (17,424 proteins, downloaded on June 14, 2018) using the following parameters: peptide and fragment mass tolerance were 10 ppm and 0.6 Da, respectively, with two miscleavages, as described previously (26). The oxidation of methionine (15.995 Da) and deamidation of asparagine and glutamine (0.984 Da) were considered as variable modifications. TMT labeling of the N-termini of peptides as well as lysine (229.163 Da) and cysteine carbamidomethylation (57.021 Da) were considered as static modifications. Relative quantitation using TMT reporter ions was performed using high-quality MS3 spectra. A percolator algorithm was used to determine the false discovery rate, and only peptides with a false discovery rate  ≤0.01 were considered for further analysis.

Log-transformed fold-change data were lowess-normalized using StatsModels (statsmodels.org), and the correlations were measured using the ordinary least squares linear regression method from SciPy (scipy.org). The sorted heat map, stacked bar charts of the log2 fold changes, and related visualization were generated by using Matplotlib (matplotlib.org). Proteins were clustered using the k-means algorithm, and the separation analysis was performed using t-distributed Stochastic Neighbor Embedding algorithm from the scikit-learn machine learning library (scikit-learn.org). Cluster maps were generated using seaborn (seaborn.pydata.org). Clustering analysis compared protein’s abundance relative to naïve (untreated) 0-h, 0-Se sample and 4, 8, and 20 h after stimulation with LPS. In each of the biological triplicates, two data points per protein were used for clustering. Values from the two replicates were averaged for drawing the curves in Figure 2*A*. Proteins identified in at least two replicates and with at least one unique peptide were included in this analysis. Kinetic deltagram algorithm was developed by Salis Lab at the Penn State University (salislab.net) to visualize fold-change trends across different time points, as well as identify the window of most notable change, and was implemented in Python. Functional data analysis was carried out using ingenuity pathway analysis (51), database for annotation, visualization and integrated discovery (52), and gene set enrichment analysis (53) for pathway enrichment.

### Sample preparation for metabolite acquisition and data analysis

Metabolites were extracted from harvested BMDMs in prechilled 80% (v/v) methanol. Samples were snap-frozen in liquid nitrogen, vortexed, and centrifuged for 20 min at 20,000*g* at 4 °C. The supernatant was dried using a SpeedVac and resuspended in 3% aqueous methanol containing 100-μM chlorpropamide as an internal standard. All samples were acquired in biological triplicate with a randomized sample order. 10 μl aliquots of each sample were analyzed using LC-MS.

Metabolites were analyzed using reverse-phase UHPLC (C_18_ Hydro-RP column; Phenomenex) coupled to an Exactive Plus Orbitrap MS (Thermo Fisher Scientific). A linear gradient from 3 to 100 (v/v) % methanol for 25 min was achieved with 3% methanol, 10-mM tributylamine, 15-mM acetic acid (solvent A) and 100% methanol (solvent B) (54). Mass spectra were acquired in a negative-ion mode, with a scan range of 85 to 1000 *m/z* with a resolution of 140,000 at *m/z* 200. A total of 47 metabolites were profiled across five time points of the experiment (0, 2, 4, 8, and 20 h) in biological triplicate. Individual metabolite extracted-ion chromatograms were used to determine the integrated peak areas. All metabolite peak areas were normalized to the chlorpropamide, and metabolite abundance was calculated relative to that derived from naive zero-hour samples. An in-house library generated from 292 authentic metabolite standards with experimentally observed accurate *m/z* values and retention times aided in the identification of metabolites (55).

### Western immunoblot

Harvested cell pellets were resuspended in the mammalian protein extraction reagent (Thermo Fisher Scientific) containing protease inhibitor mixture (Roche Applied Science) and 5-mM sodium orthovanadate (Sigma), incubated on ice for 20 min, and vortexed for 10 min followed by centrifugation for 25 min at 20,000*g* at 4 °C. The protein concentration was determined using the bicinchoninic acid protein assay kit (Thermo Fisher Scientific). Proteins were resolved using a 12.5% (% T) SDS-PAGE and transferred onto a nitrocellulose membrane. The membranes were blocked with 7% (w/v) skim milk and probed with antibodies: anti-Sdha (1:2000; Cell Signaling Technology), anti-SDHB (1:4000; Proteintech), anti-Pkm (1:10,000; Cell Signaling Technology), anti-pPkm (1: 10,000; Cell Signaling Technology), anti-Shpk (Carkl) (1:2500; MyBioSource), and anti-β-actin (1: 25,000; Fitzgerald). Data from three independent experiments were used in densitometric analysis, whereas only a representative Western blot is shown for brevity.

## Data availability

The mass spectrometry proteomics data generated in this study have been deposited at the ProteomeXchange Consortium *via* the PRIDE (56) partner repository with the dataset identifier PXD023005.

## Supporting information

This article contains supporting information.

## Conflict of interest

The authors declare that they have no conflicts of interest with the contents of this article.

## Supporting information

Supplemental Figures S1–S6

Supplementary File 1 **Database search and protein identification.** Database search results of protein identification with the UniProt accession numbers, protein description, sum pep score (obtained from Proteome Discoverer, version 2.2), sequence coverage, number of peptides, PSMs, unique peptides, amino acids, molecular weight, calculated pI, gene symbol, and quantification measurements of each proteins are provided.
