# Supplementary material for: Selenium-dependent metabolic reprogramming during inflammation and resolution
Source: J Biol Chem. 2021 Feb 11;296:100410. doi: 10.1016/j.jbc.2021.100410 (PMC7966868; doi:10.1016/j.jbc.2021.100410)
Supplement: Supplemental Figures S1–S6 [file mmc1.pdf]

SUPPORTING INFORMATION

Figure S1. Effect of LPS on the expression of prototypical genes in BMDMs. LPS-dependent expression of prototypical proteins in BMDMs by proteomic studies post 20 h. Fold changes in expression were calculated relative to unstimulated cells. For these studies, BMDMs cultured in the absence of Se were used. Mean of n= 3 independent experiments per time point were used.

Figure S2. **Clustering and functional analysis of Se-dependent effects of LPS in BMDMs.** Protein cluster enrichment 1 to 7 enrichment of post-LPS proteome and their regulation kinetics. In column **a** represent post-LPS proteome and their expression kinetics is depicted in columns **b** and **c**. The red and blue colors in columns **b** and **c** depict modulation with Se and without Se, respectively. The black patches in column **c** highlights the time interval showing most notable changes in kinetics upon stimulation with LPS. Functional analysis depicting specific metabolic pathways enriched within each cluster is shown in column **d**.

Figure S3. **Modulation of the mitochondrial proteome changes by Se supplementation (as sodium selenite, 250 nM) positively impacts the TCA cycle and OXPHOS related proteins. a.** The mitochondrial proteome was classified into six clusters based on the kinetic regulation of protein expression upon LPS stimulation. Kinetic regulation is depicted in column **b** and **c**. Pathway analysis identified the specific metabolic pathways enriched within respective cluster are depicted in column **d**.

Figure S4. **Differential modulation of cytosolic ROS (a) and mitochondrial ROS (b) in**

BMDMs by Se supplementation following LPS stimulation. BMDMs were isolated from WT mice and cultured in media with or without the presence of selenite (250 nM). BMDMs were stimulated with 100ng/ml LPS. After 1hr stimulation, LPS containing media were removed and replaced with fresh media and cells were continuously cultured for indicated time periods. Following stimulation, BMDMs were then stained for 30 min with CellROX Deep Red (500 nM) flow cytometry stain for cytosolic ROS (**panel A**) or MitoSOX Red stain (5 uM) for mitochondrial ROS (**panel B**), followed by 15 mins of the dead cell staining with propidium iodide (PI) or 7-aminoactinomycin D (7-AAD), respectively. Data shown are n= 3 per group ± SEM; *p<0.05.

Figure S5. Selenoprotein-dependent changes in glycolytic metabolites in WT and *Trsp*$^{\mathbf{fl/fl}}$*LysM*$^{\mathbf{Cre}}$ BMDMs. BMDMs isolated from WT and *Trsp*$^{fl/fl}$*LysM*^{Cre} mice were stimulated for 2-20 h with LPS. Metabolites were isolated and subjected to targeted metabolomics. Temporal regulation of glycolytic metabolites was analyzed in BMDMs. Phosphoenol pyruvate and pyruvate, was observed to be distinctly different in Se supplemented WT when compared to *Trsp*$^{fl/fl}$*LysM*^{Cre} BMDMs suggesting a causal association with selenoproteins expression. For each time point upon LPS stimulation, mean of three independent experiments (± SEM) were considered for each genotype.

Figure S6. Selenoprotein-dependent changes in PPP metabolites in WT and *Trsp*$^{\mathbf{fl/fl}}$ *LysM*$^{\mathbf{Cre}}$ BMDMs post LPS. Temporal regulation of PPP metabolites, especially sedoheptulose-1/7- phosphate was observed to be distinctly different in Se supplemented WT BMDMs compared to *Trsp*$^{fl/fl}$*LysM*^{Cre} BMDMs suggesting their modulation to be causally associated with selenoprotein expression. For each time point upon LPS

stimulation, mean of three independent experiments (± SEM) were considered for each genotype.

Supplementary File 1. Database search and protein identification. Database search results of protein identification with Uniprot accession numbers, protein description, sum pep score (obtained from Proteome Discoverer Ver2.2), sequence coverage, number of peptides, PSMs, unique peptides, amino acids, molecular weight, calculated pI, gene symbol, and quantification measurements of each proteins are provided.

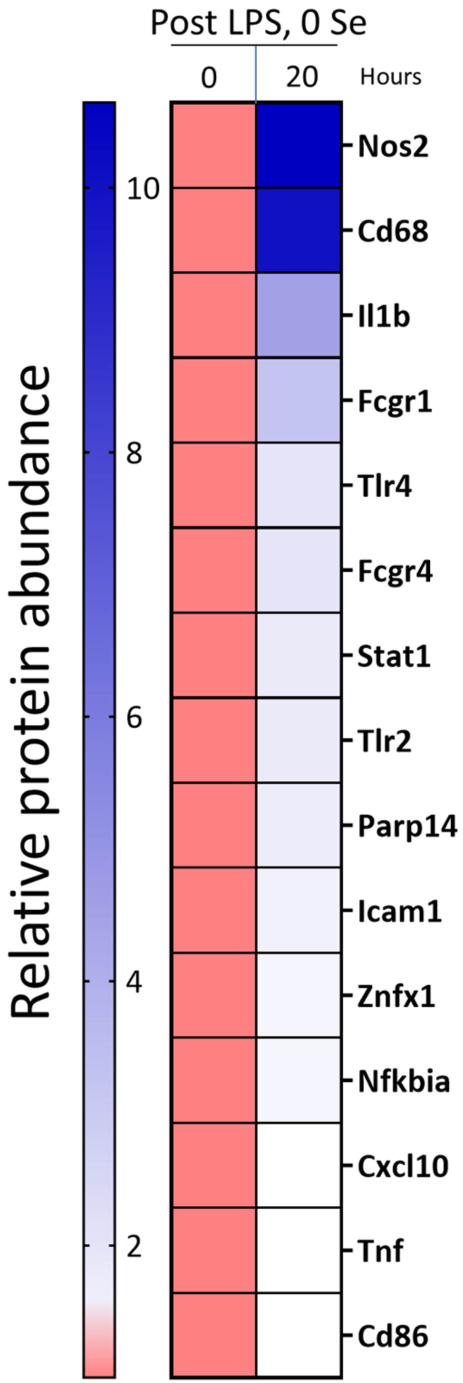

Supplementary Figure S1

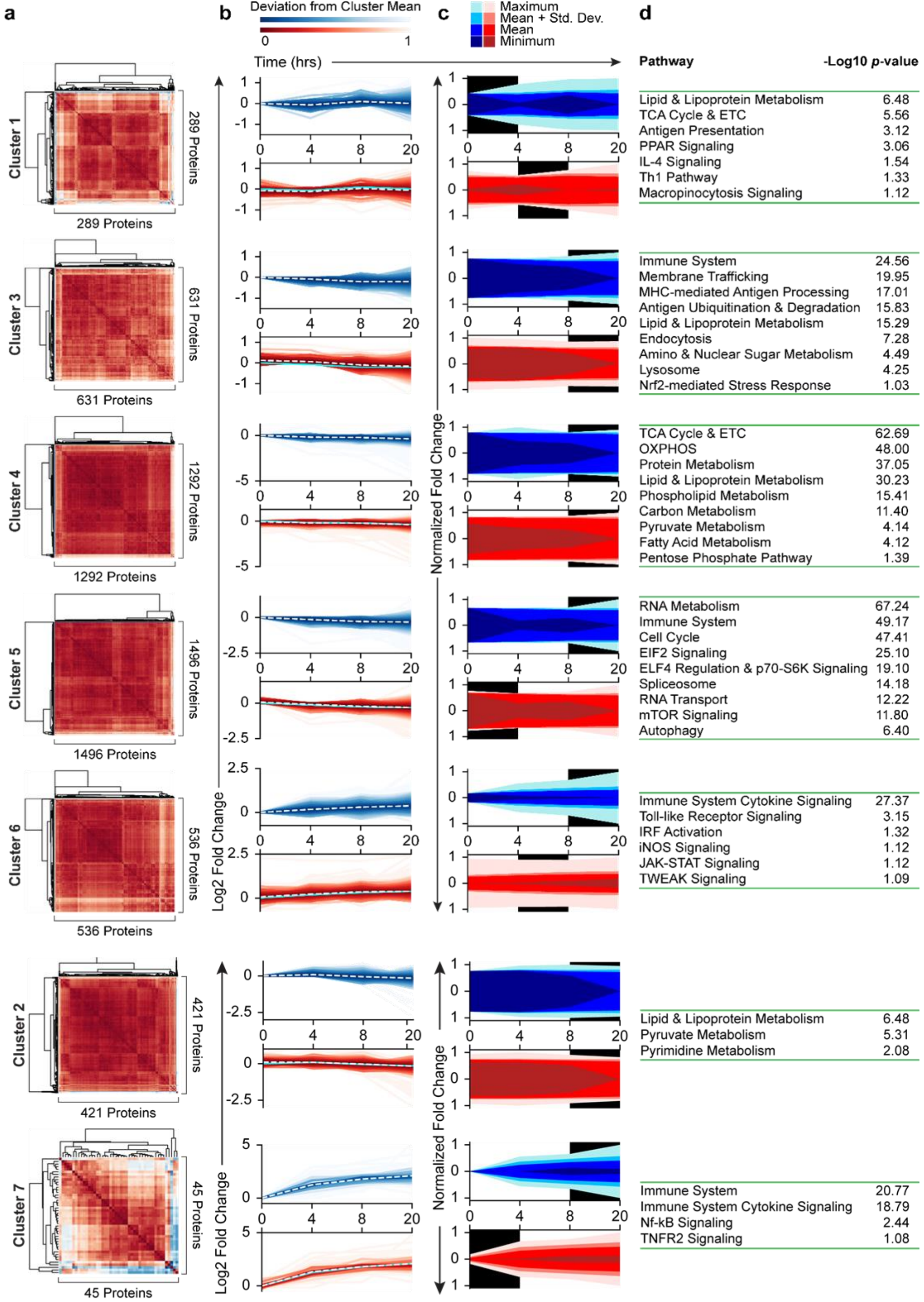

Supplementary Figure S2

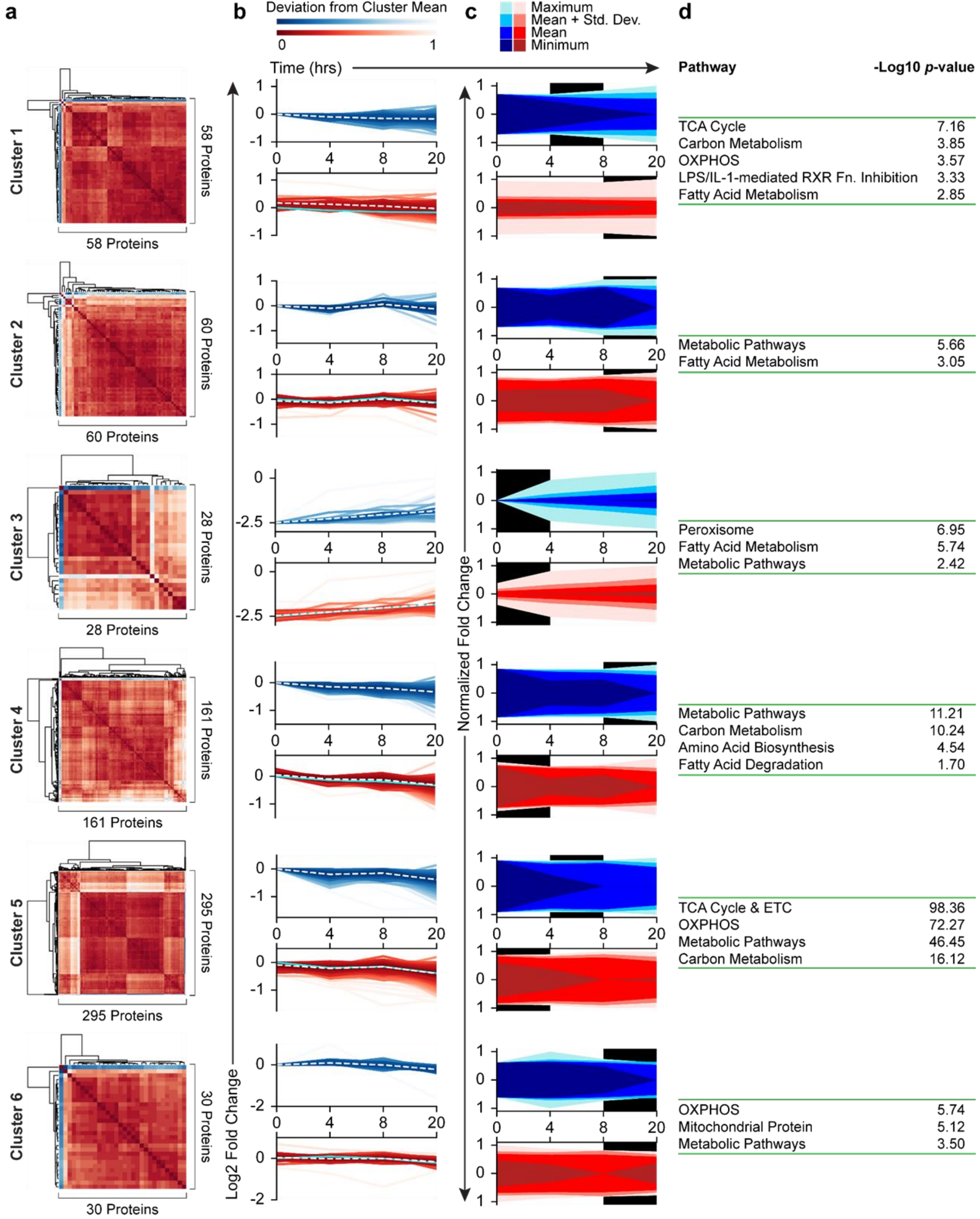

Supplementary Figure S3

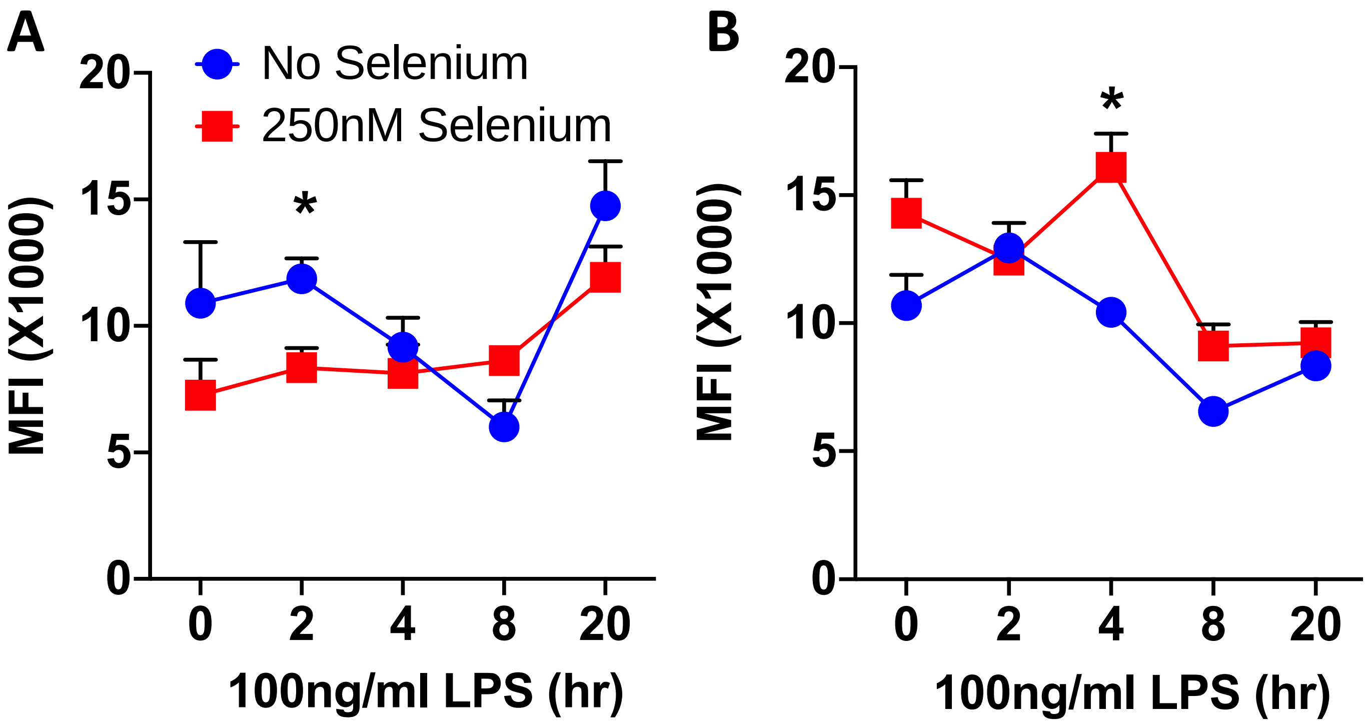

Supplementary Figure S4

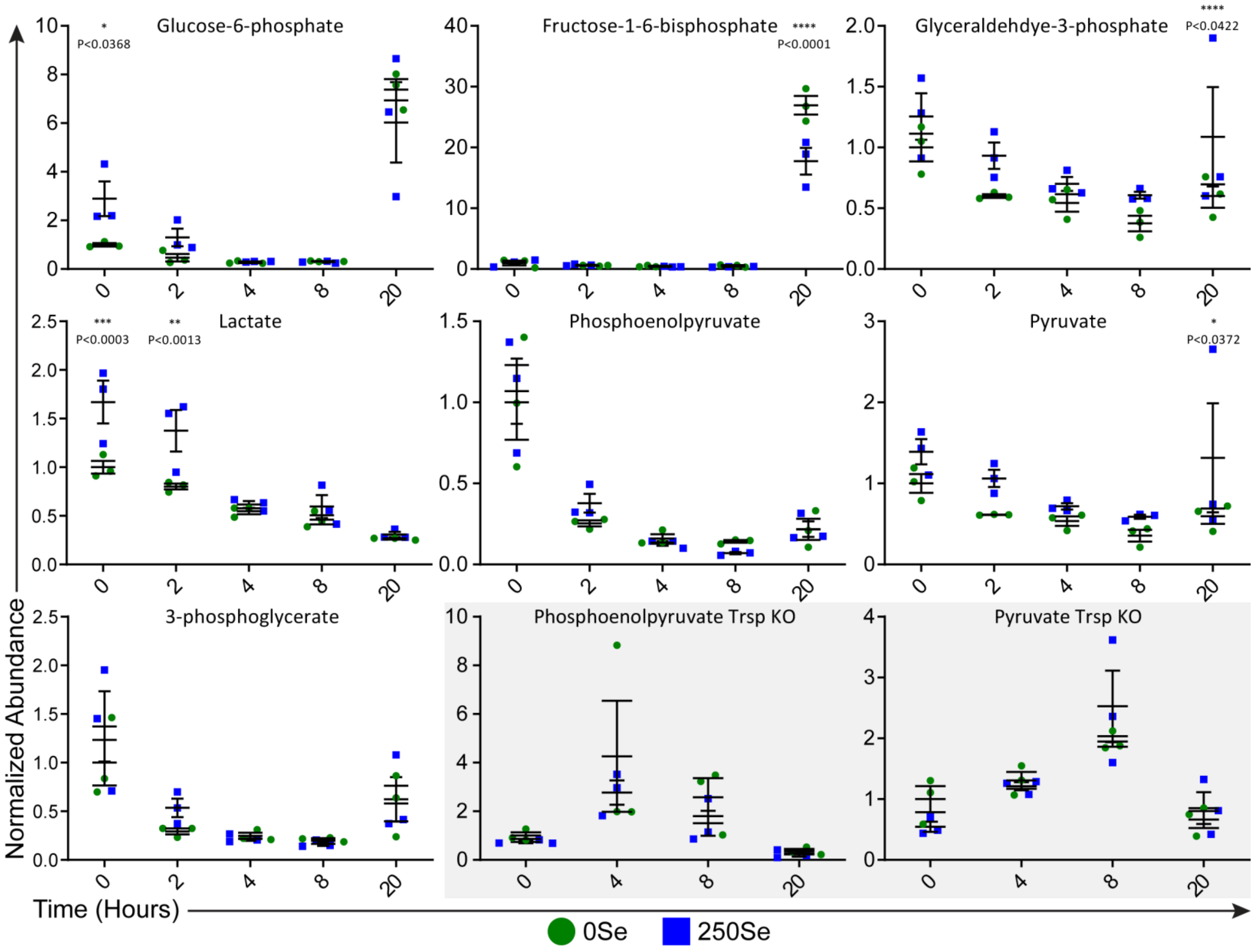

Supplementary Figure S5

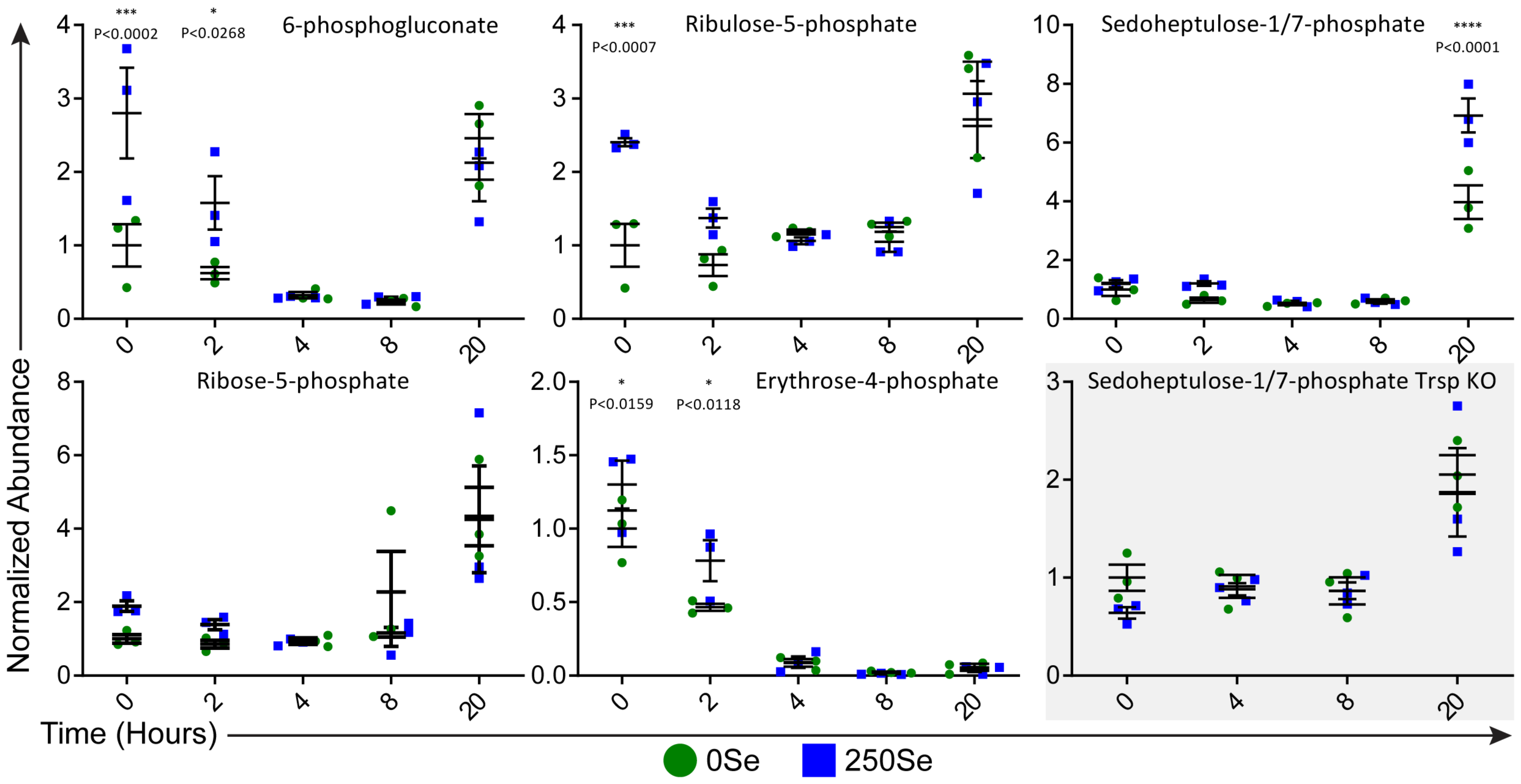

Supplementary Figure S6

Supplementary Table 1. Mass spectrometry and tandem mass tag (TMT) sample designations

Sample ID	Set	TMT Channel
0hr 0Se, Replicate #1	A	126
0hr 250Se, Replicate #1	A	127N
4hr 0Se, Replicate #1	A	127C
4hr 250Se, Replicate #1	A	128N
8hr 0Se, Replicate #1	A	128C
8hr 250Se, Replicate #1	A	129N
20hr 0Se, Replicate #1	A	129C
20hr 250Se, Replicate #1	A	130N
0hr 0Se, Replicate #2	B	126
0hr 250Se, Replicate #2	B	127N
4hr 0Se, Replicate #2	B	127C
4hr 250Se, Replicate #2	B	128N
8hr 0Se, Replicate #2	B	128C
8hr 250Se, Replicate #2	B	129N
20hr 0Se, Replicate #2	B	130C
20hr 250Se, Replicate #2	B	131
0hr 0Se, Replicate #3	C	126
0hr 250Se, Replicate #3	C	127N
4hr 0Se, Replicate #3	C	127C
4hr 250Se, Replicate #3	C	128N
8hr 0Se, Replicate #3	C	129C
8hr 250Se, Replicate #3	C	130N
20hr 0Se, Replicate #3	C	130C
20hr 250Se, Replicate #3	C	131
